# Mechanically Robust, Softening Shape Memory Polymer Probes for Intracortical Recording

**DOI:** 10.3390/mi11060619

**Published:** 2020-06-25

**Authors:** Allison M. Stiller, Joshua O. Usoro, Jennifer Lawson, Betsiti Araya, María Alejandra González-González, Vindhya R. Danda, Walter E. Voit, Bryan J. Black, Joseph J. Pancrazio

**Affiliations:** 1 Department of Bioengineering, The University of Texas at Dallas, Richardson, TX 75080, USA; Joshua.usoro@utdallas.edu (J.O.U.); jxl125231@utdallas.edu (J.L.); betsiti.araya@utdallas.edu (B.A.); walter.voit@utdallas.edu (W.E.V.); bjb140530@utdallas.edu (B.J.B.); joseph.pancrazio@utdallas.edu (J.J.P.); 2 Department of Biomedical Engineering, University of Houston, Houston, TX 77004, USA; magonzalezg@uh.edu; 3 Qualia, Inc., Dallas, TX 75252, USA; vindhya@qualiamedical.com; 4 Department of Materials Science and Engineering, The University of Texas at Dallas, Richardson, TX 75080, USA

**Keywords:** intracortical microelectrode arrays, shape memory polymer, softening, robust

## Abstract

While intracortical microelectrode arrays (MEAs) may be useful in a variety of basic and clinical scenarios, their implementation is hindered by a variety of factors, many of which are related to the stiff material composition of the device. MEAs are often fabricated from high modulus materials such as silicon, leaving devices vulnerable to brittle fracture and thus complicating device fabrication and handling. For this reason, polymer-based devices are being heavily investigated; however, their implementation is often difficult due to mechanical instability that requires insertion aids during implantation. In this study, we design and fabricate intracortical MEAs from a shape memory polymer (SMP) substrate that remains stiff at room temperature but softens to 20 MPa after implantation, therefore allowing the device to be implanted without aids. We demonstrate chronic recordings and electrochemical measurements for 16 weeks in rat cortex and show that the devices are robust to physical deformation, therefore making them advantageous for surgical implementation.

## 1. Introduction

Intracortical microelectrode arrays (MEAs) are devices that can be implanted in brain tissue to stimulate or record electrical activity from surrounding neural populations [1], making them important tools for investigating the function of the nervous system [2]. Additionally, intracortical recording MEAs have been used as critical components in brain–machine interfaces (BMIs) [3,4,5], which may be used to restore or replace loss of motor function in patients suffering from paralysis, limb loss, or neurodegenerative disorders. Despite the promise of MEA technology, the clinical adoption of these devices has been limited for many reasons, several of which are associated with stiff material composition. Commercially available devices are often fabricated from high modulus materials, such as silicon, to leverage reproducible photolithography techniques. However, this material choice also results in structures that are brittle due to the small device dimensions necessary to mitigate a severe chronic neuroinflammatory response, which may contribute to behavioral deficits [6]. This fragility renders devices vulnerable to fracture during fabrication [7] and handling [8,9]. Even after implantation, the device may be susceptible to breakage or cracking [10] due to the tethering forces caused by constant micromotion of the brain [11]. This effect may be exacerbated by the high degree of mechanical mismatch between the implanted device and the surrounding tissue, which creates a constant source of mechanical strain at the brain–device interface [12,13,14]. Because of this, many groups are investigating the potential use of robust, yet softer, polymer materials for intracortical device fabrication.

Unfortunately, there are many inherent issues associated with fabricating functional devices from soft materials. From a mechanical standpoint, polymer-based devices may lack the structural rigidity required to successfully implant in brain tissue if they are fabricated with small cross-sectional dimensions. To alleviate this issue, there have been demonstrations of the use of insertion guides to aid in implantation [15], but implementation of these aids may complicate surgical use. Others have addressed structural instability with stiffening coatings that dissolve after implantation [16,17,18], but these may increase the footprint of the device during implantation, thus increasing the risk of disrupting vasculature. Additionally, while many polymers such as SU-8 and Parylene C exhibit an elastic modulus of 1–5 GPa, approximately 2–3 orders of magnitude lower than that of materials commonly used for intracortical device fabrication, this is still 5–6 orders of magnitude higher than the estimated modulus of brain tissue (~0.5–10 kPa [19,20]). With these drawbacks in mind, there is motivation to design a robust polymer-based device that can be handled and implanted using existing surgical techniques, but that exhibits material properties that more effectively bridge mechanical mismatch with the brain.

Our group has previously investigated the use of shape memory polymers (SMPs) for fabrication of intracortical devices [21]. SMPs are a class of material that are unique in their ability to undergo dynamic changes in material properties in response to stimuli from the environment [22,23,24]. Specifically, we used a thiol-ene/acrylate formulation softened from 2 GPa in dry, room temperature conditions to ~300 MPa in wet, body-temperature conditions. This range ensured that devices could maintain structural stability during implantation, but soften by an order of magnitude in a few minutes after making contact with the brain tissue. This thiol-ene/acrylate SMP softens primarily due to an increase in temperature and minimally due to water absorption (<3% by weight). In this study, we used photolithography to create reproducible SMP based devices with the ability to soften to 20 MPa after implantation, an order of magnitude softer than our previously reported functional softening biolectronics. We demonstrate that these devices are capable of intracortical recording, show consistent electrical performance after substantial mechanical perturbation, and induce a tissue response consistent with similarly sized silicon shanks. Furthermore, we provide pilot data indicative of a reduced behavioral deficit associated with the SMP based devices.

## 2. Materials and Methods

### 2.1. Polymer Preparation and Device Fabrication

**Figure 1:**
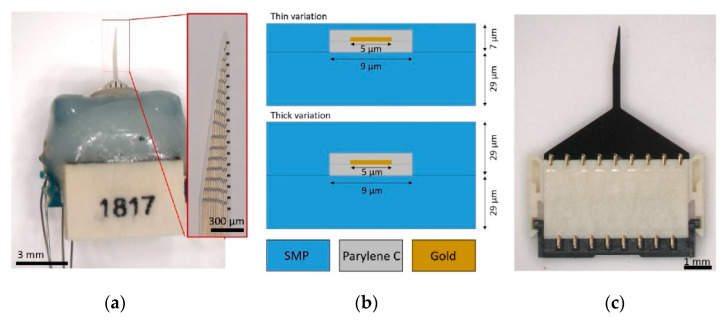
SMP and silicon device design. (**a**) Optical image of a packaged SMP device with the red inset depicting 16 electrode sites on an SMP device (the electrodes appear to be black due to the SIROF coating). The Omnetics connector is sealed off with epoxy (grey encapsulation material). (**b**) Representative cross-sectional view of a gold trace and coaxial Parylene C insulation in both device variations. (**c**) A size-matched non-functional silicon device mounted in a zero insertion force (ZIF) connector.

The SMP substrate was prepared as previously described [25] and material properties were determined using dynamic mechanical analysis (DMA), a methodology that applies cyclic, tensile loading to samples while changing the temperature in dry or aqueous conditions. DMA results indicated that the SMP exhibited an elastic modulus of 2 GPa in dry, room-temperature conditions and 20 MPa in soaked, body-temperature conditions. The pre-polymer solution was prepared using 1,3,5-triallyl-1,3,5-triazine-2,4,6 (1H,3H,5H)-trione (TATATO), trimethylolpropane tris(3-mercaptopropionate) (TMTMP), and (2-(3-mercaptopropionyloxy) ethyl) isocyanurate (TMICN) at molar ratios of 0.5:0.45:0.05, respectively, with 0.1 weight percent 2,2-dimethoxy-2-phenylacetophenone (DMPA), a photoinitiator. The solution was spun onto 4 inch silicon wafers (test grade, laser scribed, P-type, boron-doped wafers) at 600 rpm for 30 s and subsequently cured under UV light for 3 min at 254 nm and 1 h at 365 nm. The polymerized samples were then transferred to an oven for an overnight vacuum bake at 120 °C. This spin coating process yielded a 29 µm thick SMP layer on the silicon wafers. All processing, with the exception of SMP spin-coating and device release, was done in a Class 10,000 cleanroom at the University of Texas at Dallas (UTD). All photomasks needed for device fabrication were designed using AutoCAD and fabricated in the above cleanroom using a Heidelberg DWL66 laser lithography system (Heidelberg, Germany). The SMP layer was cleaned with deionized water followed by a nitrogen dry and dehydration bake at 115 °C in a vacuum oven for 15 min to promote adhesion. A 1 µm layer of Parylene C was then deposited on the bottom SMP layer to act as an insulator, using a SCS PDS 2010 coater (Specialty Coating Systems, Indianapolis, IN, USA). Gold interconnects (5 µm wide) were patterned by liftoff lithography using nLOF photoresist and low stress silicon nitride (900 mT, 150 °C, 100 W with SiH_4_, NH_3_, H_3_, and N_2_ gases at 280, 4600, and 200 sccm, respectively). A 400 nm gold thin film was electro-beam evaporated on the wafer. A secondary 1 µm layer of Parylene C was deposited, and the wafers were patterned using standard lithography techniques and etched using oxygen plasma to form a coaxial layer around the gold traces. The wafers then went through a dehydration bake at 115 °C for 15 min and immediately received a 7 μm thick top coat of the SMP using spin parameters of 1200 rpm for 2 min. They were then returned to the cleanroom for additional processing. Electrode sites, bond pads, and the device outline were patterned using standard photolithography methods. A hard mask of ~200 nm low stress silicon nitride was used to perform the plasma etching of SMP to open the electrode sites and bond pads and create the device outline. After etching, the hard mask was stripped off using hydrofluoric acid, and electrode sites (18 × 10 µm) were patterned with liftoff lithography using AZ400K solvent at room temperature. Next, the electrode sites received sputtered iridium oxide film (SIROF) to decrease electrode site impedance. First, a 20–50 µm layer of titanium was deposited (200 V, 4 Torr, with Ar flow at 50 sccm) to act as an adhesion layer for the SIROF. Next, a 160–180 µm layer of iridium oxide was sputtered onto the electrode sites (100 V, 30 Torr, with Ar, O_2_, and 2% H_2_/98% Ar at 15, 20, and 10 sccm, respectively). After the fabrication was complete, wafers were soaked in DI water in a 37 °C oven until individual devices released from the wafers. Released devices were assembled with packaging with Omnetics connectors (A79040-001, Omnetics Connectors Corporation, Minneapolis, MN, USA) by Qualia Labs, Inc. (Figure 1a). A thicker variation of the SMP devices were also fabricated for the purposes of mechanical testing. In this case, all fabrication steps were identical with the exception of a 29 µm thick top SMP layer in place of the 7 µm layer (Figure 1b). Size-matched non-functional silicon devices were also fabricated in UTD cleanroom facilities using standard photolithography techniques (Figure 1c).

### 2.2. Surgical Implantation

All animal handling, housing, and surgical procedures were approved by the University of Texas Institutional Animal Care and Use Committee. Male, Long Evans rats (n = 8, Charles River) weighing 600–800 g received bilateral implants of functional SMP devices and non-functional silicon shanks.

Surgical methods followed those previously reported [21]. Briefly, animals were anesthetized with an intraperitoneal injection of a ketamine cocktail (ketamine (65 mg/kg), xylazine (13.33 mg/kg), and acepromazine (1.5 mg/kg)) followed by an intramuscular injection of atropine sulfate (0.05 mg/kg) to counteract depressive cardiac effects of the ketamine and a subcutaneous injection of dexamethasone (2 mg/kg) to help mitigate post-operative inflammation. When unconscious, the animal’s head was shaved and then mounted in a stereotaxic frame with a nose cone delivering 2% isoflurane in 100% oxygen for maintenance of the anesthetic plane. Ophthalmic ointment was applied to the eyes to retain moisture and prevent drying during the surgery. The scalp was cleaned alternating with 10% iodine solution and 70% ethanol wipes three times and then injected with 0.4 mL of 0.5% lidocaine to numb the area. An incision was made using a surgical blade down the midline of the scalp, and hemostatic forceps were used to hold the skin away from the skull. The surface of the skull was thoroughly cleaned and roughened to promote adhesion of the head cap.

**Figure 2:**
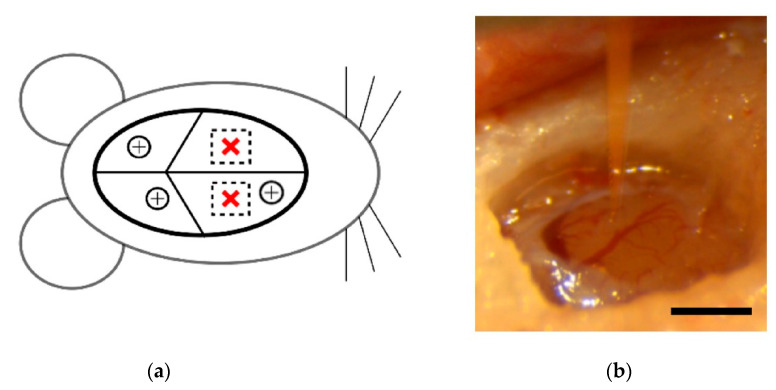
Device implantation. (**a**) Schematic depicting bilateral implant locations (red crosses) in rat brain. The circled plus signs indicate placement of stainless steel screws. (**b**) SMP shank held over a craniotomy with resected dura immediately before implantation. Scale bar = 1 mm.

First, a surgical drill was used to create three small holes in the skull for positioning of stainless steel screws. These screws provided mechanical support for the head cap but also served as ground and reference connections for external wiring on the functional SMP device. Next, 1–2 mm craniotomies were drilled over each motor cortex, centered approximately 2.5 mm rostral and 2.5 mm lateral from bregma (Figure 2a). The dura was resected over one craniotomy and a device was positioned over the center (Figure 2b). The device was implanted at a speed of 1 mm/s using a hydraulic micropositioner (Kopf Instruments, Tujunga, CA, USA) to a depth of 2 mm. The dura was then replaced with collagen-based grafts (Biodesign Dural Graft, Cook Medical, Bloomington, IN, USA). After the grafts were rehydrated by the surrounding cerebrospinal fluid, a layer of Gluture (World Precision Instruments, Sarasota, FL, USA), a biocompatible cyanoacrylate, was used to seal off the craniotomy. After implantation of the SMP device, ground and reference wires were wrapped around stainless steel screws while waiting for the cyanoacrylate to dry. Finally, a small amount of dental cement was applied around the device base in order to stabilize it during implantation of the second device. This process was then repeated for the second device in the contralateral cortex. Following both implantations, the head cap was built up to encapsulate the devices, leaving only the Omnetics connector visible. The skin behind the implantation sites was closed using surgical staples and left in place for 10 days following the operation. The animal then received 2–3 mL of sterile saline for hydration, a subcutaneous injection of sustained release buprenorphine SR LAB (ZooPharm, Windsor, CO, USA), and an intramuscular injection of cefazolin antibiotic (5 mg/kg). The animal received a follow-up shot of buprenorphine 72 h after surgery.

### 2.3. Electrophysiological Recordings and Electrochemistry

We performed weekly electrophysiological recordings and electrochemistry on anesthetized animals (1–2% isoflurane) starting immediately after surgery, at Week 0, for 16 weeks. Spontaneous wideband data were recorded for 10 min at 40 kHz with functional SMP devices using a 16-channel Plexon headstage and Omniplex acquisition system (Plexon, Inc., Dallas, TX, USA). A four-pole Butterworth high pass filter with a cutoff frequency of 250 Hz was applied to the wideband data, and spikes were manually sorted using 2D principal component space after identifying potential spikes that crossed a threshold of –4 σ (based on the root mean square (RMS) of the filtered signal). Units were only considered for analysis if they contained at least 100 spike waveforms. A custom MATLAB code was used to extract mean peak-to-peak voltage (Vpp), RMS noise, and signal to noise ratio (SNR) calculated by dividing Vpp by two times the RMS noise of the associated channel.

Electrochemical impedance spectroscopy (EIS) was performed using a model 604E series electrochemical analyzer/workstation (CH Instruments, Inc., Austin, TX, USA). An 18-pin dual strip Nano-D female connector (NSD-18-WD-18.0-C-GS, Omnetics Connector Corporation, Minneapolis, MN, USA) was attached to the SMP Omnetics connector to interface with the system. The device wires wrapped around the stainless steel screws were shorted together to act as a reference electrode and the SIROF-coated electrodes served as the working electrodes. A 10 mV RMS sinusoidal signal ranging from 100 kHz to 1 Hz was sent to the device, and current was recorded 3 times per decade of frequency. CH Instruments software calculated impedance magnitude, and a custom MATLAB code extracted real impedance values at each point.

### 2.4. Behavioral Testing

**Figure 3:**
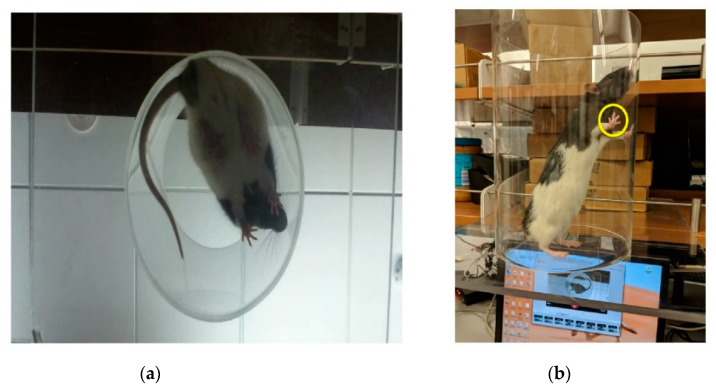
Cylinder test setup. (**a**) The rat is filmed from underneath as it rears up. (**b**) The rat rears up to explore its surroundings. The yellow circle indicates a paw touch against the cylinder.

In an effort to compare the potential behavioral changes associated with implantation of SMP and silicon shanks, we conducted pilot experiments using the cylinder test. The cylinder test takes advantage of rodents’ natural affinity towards exploring novel environments to determine changes in limb-use asymmetry and overall exploratory behavior over time [26,27]. Rats were placed in a transparent plastic cylinder and were allowed to freely explore for 3 min while being filmed from underneath through a clear acrylic sheet (Figure 3a). The number of times the left paw, right paw, or both paws, were used to initially reach out and touch the walls of the cylinder was manually counted from the video (Figure 3b). Initial paw preference was determined by baseline measurements that were taken prior to surgical implantation. Cylinder testing was performed weekly beginning one week post-implantation. For analysis, rats were grouped based on initial paw preference and the type of device that was implanted contralateral to that paw. The percent use of each paw was then tracked over the 16-week study.

### 2.5. Immunohistochemistry and Analysis

After 16 weeks, animals were sacrificed with an intraperitoneal injection of sodium pentobarbital (200 mg/kg). Tissue fixation was performed by transcardial perfusion with 4% paraformaldehyde (PFA). Whole brains were dissected, sectioned into hemispheres, and stored in PBS + 0.5% sodium azide at 4 °C. For cryoprotection, brains were incubated overnight in 15% sucrose (Sigma-Aldrich, St. Louis, MO, USA) solution in PBS and the following night in a 30% sucrose solution. The samples were then transferred to cryomolds filled with optimal cutting temperature compound (OCT, Sekura Finetek, Torrance, CA, USA). Flash freezing was then achieved by submerging cryomolds containing individual hemispheres into dry ice plus isopropyl alcohol. Once the OCT-embedded samples were frozen, they were placed in a −20 °C freezer until sectioned. Individual brain hemispheres were horizontally sectioned into 50 µm slices using a Leica CM3050 S cryostat and adsorbed onto charged slides (Fisher Scientific, Waltham, MA, USA) for staining.

Cryosections were permeabilized using 0.5% Triton X-100 (Sigma-Aldrich, USA) in 4% normal goat serum (NGS) (Abcam, Cambridge, UK) and 1× PBS and then blocked in a solution of 0.5% TritonX, 4% NGS, and 1× PBS. Sections were then incubated with mouse anti-neuronal nuclei (NeuN, 1:200, Abcam, ab104224), chicken anti-glial fibrillary acidic protein (GFAP, 1:200, Abcam, ab4674), and rabbit anti-CD-68 (1:200, Abcam, ab125212) diluted in a solution of 4% NGS and PBS for 24 h on a rocker at 4 °C. Following primary incubation, tissue was washed 3 times for 30 min using PBS + 0.5% Triton X-100 solution and then washed with 4% NGS + PBS solution for three hours. Sections were then incubated overnight on a rocker at 4 °C with secondary antibodies: goat anti-chicken Alexa Fluor 647 (1:200, Abcam, ab150171), goat anti-rabbit Alexa Fluor 488 (1:200, Abcam, ab150077), and goat anti-mouse Alexa Fluor 555 (1:200, Abcam, ab150118), along with 0.06% concentration DAPI (0.06%, Abcam, ab228549). Sections were then washed 3 times for 10 min in PBS solution. The PBS was replaced with drops of Image-iT (Invitrogen, Camarillo, CA, USA), and then sections were sealed with coverslips using clear nail polish.

Stained slides were imaged at 10× magnification on an inverted Nikon eclipse Ti (Nikon, Tokyo, Japan) scanning confocal microscope. With the implantation site centered in the field of view, 4-color z-stack confocal images were collected across the entire tissue slice thickness in 5 µm axial steps. Care was taken to adjust laser line and sensory gains such that no saturating pixels were detected. Each laser line was scanned individually to reduce signal bleed-over. Z-stack images were then converted to a single maximum intensity projection image for analysis. Normalized GFAP and CD68 fluorescence intensities and normalized NeuN+ cell counts were carried out using boutique ImageJ [28,29] macros, as previously described [30] with minor alterations. Briefly, any regions of the image that were dark due to absence of tissue (including tears or edges) were manually excluded and converted to “not a number” (NaN) values. Shank sites were then manually defined to enable analysis within concentric contours from the site. Average mean intensity values were then automatically calculated within concentric bands of 50 µm up to 400 µm from the shank site. Lastly, based on user-defined noise tolerances, a 3 pixel Gaussian blur was applied to the NeuN channel image and cell counts were automatically collected based on local maxima detection. GFAP intensity measurements and NeuN+ cell counts were then normalized to the furthest concentric band (350–400 µm).

### 2.6. Device Physical Robustness

We tested physical robustness of functional SMP devices compared to non-functional silicon devices using electrochemical endpoints after physical deformation. For these experiments, we tested an updated version of the device that featured a thickness of 58 μm but kept all other parameters constant. These devices were also tested in a subset of animals to demonstrate improved mechanical stability during implantation as compared to the thinner design. Before device bending, we performed EIS on functional SMP devices in 1× PBS, pH 7.4, at room temperature using methods identical to those described in Section 2.3. After testing, the devices were dipped in deionized water to remove any salts that accumulated on the electrode sites during testing and allowed to dry for 24 h.

**Figure 4:**
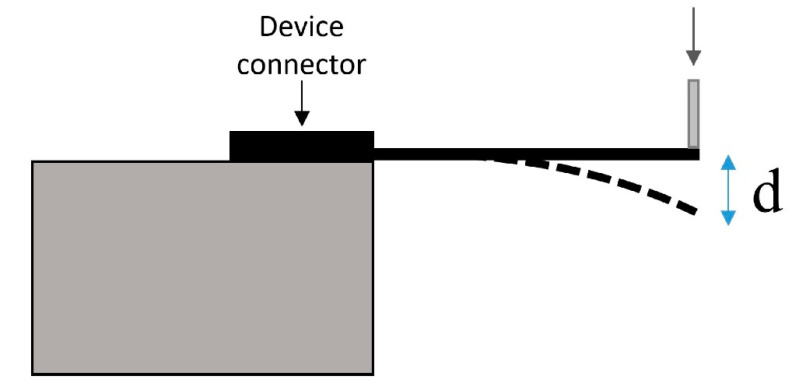
Device robustness testing setup. The device is mounted via the connector as a cantilevered beam on a stage with the entire length of the shank suspended parallel to the ground. The tip of the device is deflected downward at a speed of 1 mm/s (indicated by downward arrow) and the shank deforms accordingly (dashed line). In the case of the silicon shank, fracture occurs after a certain displacement (d). This displacement is then transiently applied in the same manner to the SMP devices.

Next, non-functional silicon devices were mounted on a stage such that the shank was parallel to the ground (Figure 4). The tip of the shank was then deflected at a speed of 1 mm/s using a custom attachment mounted to a NeuralGlider cortical neural implant inserter (Actuated Medical, Inc., Bellefonte, PA, USA). As soon as the shank broke, the system was manually stopped, and the displacement distance was recorded. This was repeated for n = 3 samples, and the average distance was recorded and used as an endpoint for deflection of the SMP devices. After drying completely, the SMP devices were mounted in an identical fashion to the silicon devices. The shank tips were displaced to the distance at which the silicon shanks broke at a speed of 1 mm/s. The SMP devices then underwent another round of EIS testing to allow for comparison of impedance magnitude values before and after deformation. Impedance magnitudes before and after physical deformation were then compared. Electrodes with an impedance magnitude greater than 1 MΩ at 1 kHz (approximately double the expected impedance) before deflection testing were excluded from analysis.

### 2.7. Statistics

All statistics were performed in MATLAB R2018b (The MathWorks, Inc., Natick, MA, USA) and OriginPro 2020b (Origin Lab, Northampton, MA, USA). Significant increases/decreases (ρ < 0.05) in electrophysiological and electrochemical data over time were determined using analysis of variance (ANOVA) tests on residuals. ANOVA tests were performed to determine significant differences (ρ < 0.05) in immunohistological outcomes between brain slices containing silicon and SMP probes. A paired t-test was used to determine significant difference (ρ < 0.05) between EIS measurements taken before and after SMP probe deflection during robustness testing.

## 3. Results

### 3.1. Single Unit Recordings and In Vivo Electrochemistry

**Figure 5:**
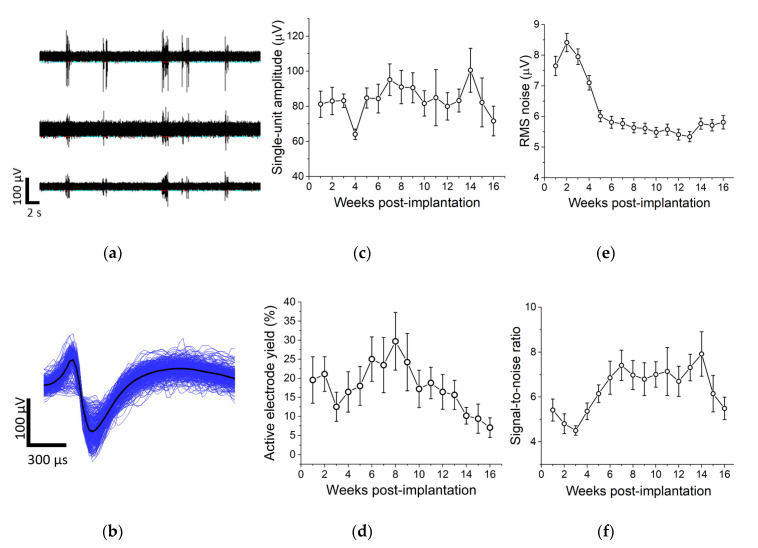
Chronic electrophysiological recordings. (**a**) Representative filtered data from three electrodes on one device. (**b**) Representative single unit activity on one electrode. We extracted measurements for single-unit amplitude (**c**), active electrode yield (**d**), RMS noise (**e**), and signal-to-noise ratio (**f**) from filtered wideband data. Linear regression analysis showed no change in single-unit amplitude over time. There was a decrease in active electrode yield (R^2^ = 0.03, ρ = 0.03) and RMS noise (R^2^ = 0.09, ρ < 0.01) and an increase in SNR (R^2^ = 0.02, ρ < 0.01) over time. Data are represented as mean ± SEM.

We performed weekly electrophysiological recordings from functional SMP devices in rat cortex and tracked single-unit amplitude, active electrode yield percentage (AEY), RMS noise, and signal-to-noise ratio (SNR) over 16 weeks (Figure 5). Results showed stable measurements for single-unit amplitude (Figure 5c), a decrease in RMS noise (Figure 5e), and an increase in signal-to-noise ratio over time (Figure 5f). However, the percentage of active electrodes, while relatively stable for the first 12–13 weeks, showed a decline in the last month of recording (Figure 5d). While one device failed to record single units during every recording session, 7 out of 8 devices exhibited active electrodes during each recording session.

**Figure 6:**
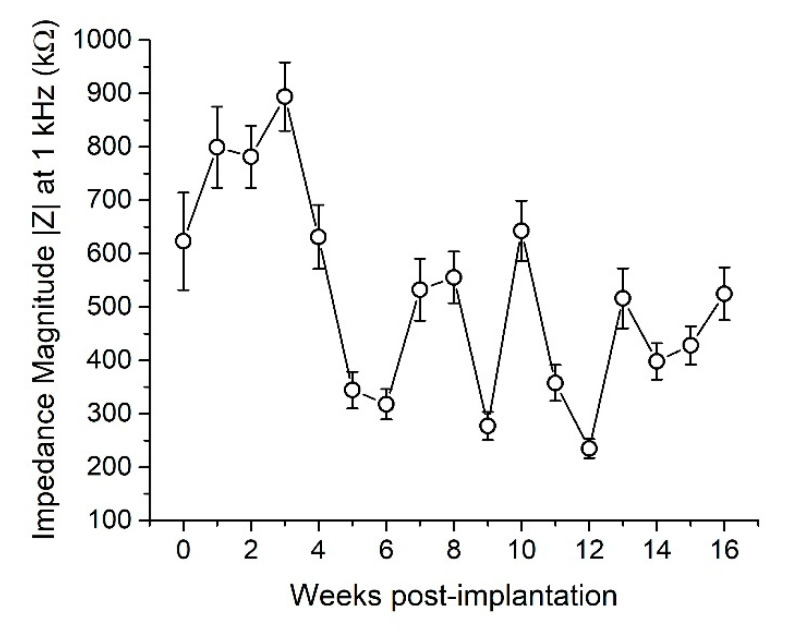
Impedance magnitude at 1 kHz over 16 weeks. Linear regression analysis shows a slight decrease in impedance over time (R^2^ = 0.03, ρ < 0.01). Data are represented as mean ± SEM.

We also performed weekly EIS measurements to assess electrode electrochemical stability. Results showed a slight decrease in impedance magnitude 1 kHz over 16 weeks with average values around 600 kΩ on Week 0 and 500 kΩ on Week 16 (Figure 6). Because of the ultra-softening nature of this SMP, we did not perform in vitro EIS on devices before implantation to premature material deformation. However, EIS measurements were taken on a small cohort of devices from the same fabrication batch to ensure impedance magnitudes were within an acceptable range (100–900 kΩ) on a majority of electrodes. In vitro measurements yielded impedance magnitudes (at 1 kHz) of ~100–250 kΩ, indicating that electrode impedance increased immediately after implantation.

### 3.2. Device Physical Robustness

**Figure 7:**
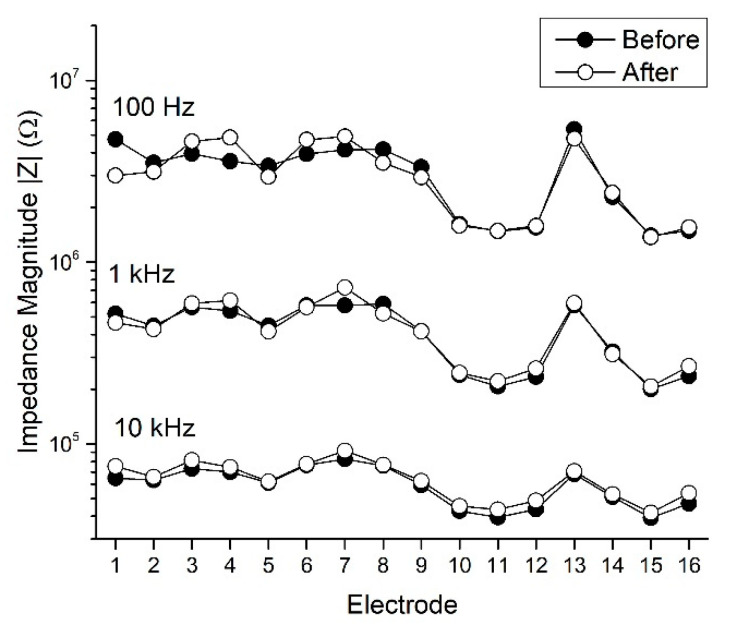
Representative EIS results from a single SMP device before and after deformation show minimal changes in impedance magnitude at 100 Hz, 1 kHz, and 10 kHz due to device deformation. The electrodes on this device exhibited an average decrease in impedance magnitude of −0.05 ± 4.2% (mean ± SEM) at 100 Hz (n = 16, ρ = 0.84), and an average increase in impedance magnitude of 2.8 ± 2.4% at 1 kHz (n = 16, ρ = 0.46) and 7.2 ± 1.2% at 10 kHz (n = 16, ρ < 0.001). The electrode layout was such that electrode 1 was located at the tip of the shank and electrode 16 was furthest up the shank.

We subjected non-functional silicon shanks to deflection until fracture and then applied the same single displacement of approximately 1.92 mm to functional SMP devices. The mechanical deformation caused a minor, yet statistically significant change in the impedance magnitude at 1 kHz across the microelectrode sites of all tested devices. The mean impedance magnitude before deformation was 539.6 ± 39.0 kΩ, rising to 583.8 ± 46.9 kΩ following deformation, a 7.6% increase (n = 31 microelectrode sites, ρ = 0.013). Similar results were observed at 10 kHz, which showed 7.5 ± 1.2% (ρ < 0.001) increases after deformation. Increases in impedance at 100 Hz, 6.9 ± 3.9% were not statistically significant. Regardless, the electrochemical characteristics of the SMP microelectrode sites exhibited tolerance to mechanical deformation (Figure 7).

### 3.3. Immunohistochemistry

**Figure 8:**
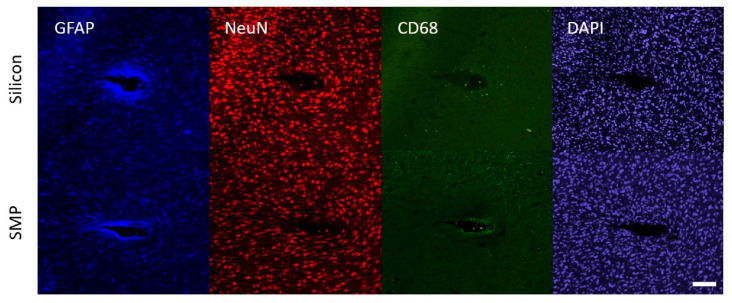
Representative immunofluorescent images for GFAP, NeuN, CD68, and DAPI stain in a middle brain slice containing a silicon device (top row) and an SMP device (bottom row). Scale bar = 100 µm.

To assess the extent of neuroinflammation after 16 weeks, we performed immunohistochemistry to stain for markers associated with three cell types commonly implicated in the chronic neuroinflammatory response: GFAP for astrocytes, NeuN for neuronal nuclei, and CD68 for activated microglia/macrophages (Figure 8). We also measured the size of the hole left behind by the explanted device in each slice. Histological results were quantified for silicon and SMP devices at three depths along the device shank. Intensity or density values for each band were normalized to the band 350–400 μm from the perimeter of the device. ANOVA results indicated no significant differences in GFAP intensity, neuronal density, and CD68 intensity between silicon and SMP shanks at any comparison point (band or depth). There was also no significant difference in hole size between slices with silicon and SMP shanks at each depth; however, the hole size at the top layer (for both device types) was significantly larger than that found at the middle or deep slices. This trend was expected due to the tapered geometry of the shanks. Similarly, for both GFAP and CD68 intensity, we observed a trend of decreased astrogliosis and microglia/macrophage activation deeper along the shank, likely also in response to decreasing cross-section of the device. There appeared to be no loss of neuronal density in any band at any depth. Separate brain slices were also stained for immunoglobulin G (IgG), a marker associated with blood–brain-barrier leakage, but ANOVA results indicated no significant differences at any comparison point.

### 3.4. Pilot Behavioral Deficit Data

**Figure 9:**
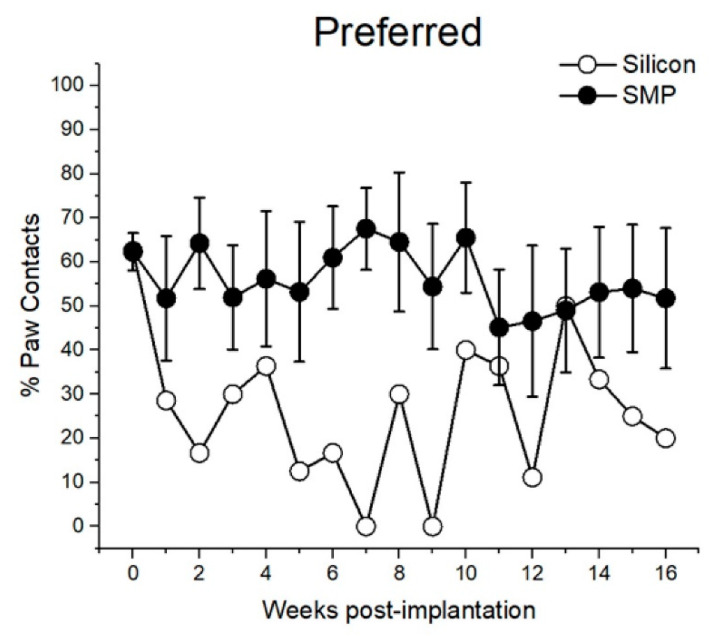
Percent of paw contacts as a function of device type implanted contralateral to the preferred paw (based on baseline data). SMP probe data are represented as mean ± SEM.

All animals were assessed weekly for motor deficit through use of the cylinder test. Their initial preferred paw was determined by majority percentage of paw contacts from baseline data taken a week before device implantation. Figure 9 demonstrates percentage of paw contacts as a function of device type implanted contralateral to the preferred paw. Unfortunately, due to randomized placement of devices, there was only one animal with a silicon device implanted contralateral to its preferred paw. However, these preliminary data suggest that animals with the SMP device implanted contralateral to their preferred paw continued using this paw, whereas the opposite was true for the animal with a silicon shank implanted contralateral to the preferred paw.

## 4. Discussion

Functional 16-channel SMP devices were implanted in rat motor cortexes for 16 weeks and their performance was tracked with weekly electrophysiological and electrochemical measurements. Within the first few weeks of implantation, active electrode yield decreased and impedance magnitude at 1 kHz increased; however, both of these trends began to reverse by week 4. Similar results were seen in our prior work [21] and may suggest onset and subsequent resolution of the acute neuroinflammatory response. While impedance magnitude remained stable for the remainder of the study, active electrode yield began dropping off in the last month of recordings. Though stable impedance values indicate that the integrity of the recording sites was likely maintained, it is possible that some degree of polymer breakdown could have contributed to decreased active electrode yield. Previous in vitro testing has validated that this variation of SMP is not hydrolytically stable and may experience degradation after chronic soaking [31]. Because of this, we are investigating a novel SMP formulation that is hydrolytically stable [31] for future chronic studies.

These updated devices were also used to demonstrate mechanical robustness in bend tests. SMP devices were able to withstand physical deformations that resulted in brittle fracture of silicon counterparts, a common issue that can make them difficult to handle during fabrication, packaging, and sterilization, and during surgical implantation. Polymers provide a clear advantage in that they may undergo large deformations but maintain integrity of electrical contacts. Furthermore, SMP-based devices may exhibit an even greater robustness to physical deformation when in the softened state due to the increase in flexibility, or decrease in stiffness, of the device. For example, the approximate stiffness of all discrete device components (gold electrode traces, Parylene C encapsulation, and SMP substrate) can be calculated using the modulus, area moment of inertia, and length of the individual components. SMP in the dry state exhibits a stiffness of approximately 1 N/m, but decreases to 0.01 N/m after softening. For comparison, an individual gold trace and coaxial Parylene C insulation exhibits a stiffness of approximately 10^−7^ (N/m) and 10^−6^ (N/m), respectively, meaning that neither the gold nor the Parylene C should inhibit robustness to physical deformation. The improved flexibility of this SMP formulation compared to that which was previously investigated (0.01 N/m versus 0.1 N/m, respectively) [21] is notable, considering the role that device stiffness may play in chronic functionality. The brain experiences continuous micromotion [11], thus warranting the need for a device that can endure constant mechanical disturbances. This need extends even further to brain macromotion, which may require even more flexibility of devices, especially in larger animal models or humans.

Additionally, given that SMP devices elicit a minimal neuroinflammatory response that is no worse than size-matched silicon devices, they may be advantageous alternatives devices for chronic use. Finally, preliminary behavioral outcomes indicated that softer devices might mitigate motor defects associated with implantation of stiff, silicon-based devices. More comprehensive analyses are required to examine the benefit of softening devices relative to behavioral measures.

## 5. Conclusions

In this work, we demonstrated fabrication and implementation of an SMP-based, 16-channel intracortical device that soften from ~2 GPa to ~20 MPa after implantation. We demonstrated an active electrode yield of 20–30% within the first 3 months of recordings with a final active electrode yield of ~10%. Weekly EIS measurements showed electrochemical stability over 16 weeks. Additionally, chronic immunohistological outcomes showed that SMP devices elicited a response no worse than bilaterally implanted silicon devices, making them good candidates for surgical implementation. Moreover, deformation testing indicated that SMP devices are mechanically robust as compared to silicon counterparts that are vulnerable to brittle fracture, further indicating that SMP offers material advantages for fabrication and handling.
